# Ralph A. Bradshaw: Scholar, leader, entrepreneur

**DOI:** 10.1096/fba.2022-00088

**Published:** 2022-10-03

**Authors:** Philip D. Stahl

**Affiliations:** ^1^ Washington University School of Medicine (Emeritus) St. Louis Missouri USA

Ralph Bradshaw, sage colleague, entrepreneur, gifted editor, and prescient scientist, recently stepped down as the Editor in Chief of *FASEB BioAdvances*. This presents the opportunity to pause and reflect on the career of an extraordinary individual, whose continuing commitment to science, science publishing, and the scholarly societies that represent and advocate for us all, illustrates the exceptional. Ralph's career reflects an unyielding dedication to those goals that we all strive to achieve—scholarship with high standards, leadership, service, and entrepreneurship.

Ralph grew up in the Boston area and attended Colby College, where he majored in chemistry; he completed his doctorate with Robert Hill in the Biochemistry Department at Duke, where his thesis work focused on protein chemistry. His postdoctoral work at Indiana University in the laboratory of Frank Gurd and the University of Washington in the laboratory of Hans Neurath allowed him to refine his repertoire of protein sequencing and analytic methodologies. P. Roy Vagelos (former CEO of Merck and former department head at Washington University) recruited Ralph to the Department of Biological Chemistry at Washington University as Roy began a highly successful renovation and rebuilding of the department after the long reign of Carl Cori. This is where I first met Ralph, as we were newly appointed assistant professors in biochemistry and physiology, respectively, and shared our interest in graduate education.

In 1973, Vagelos and colleagues at Washington University advanced a novel approach to graduate education in the biological and biomedical sciences, the Division of Biology and Biomedical Sciences (DBBS)—recently named the Roy and Diana Vagelos Division of Biology and Biomedical Sciences. The idea was based on the ongoing diversification of medical and biological research, where the traditional departmental boundaries that separated disparate “fields of research” were increasingly seen as hardened silos that suppressed innovation and reduced opportunities for graduate research. By creating programs built on common faculty interests rather than departmental affiliation, cross disciplinary opportunities for graduate research flourished—this approach in one form or another is now common among nearly all research institutions. Ralph Bradshaw played a key role in getting the DBBS off the ground—perhaps a precursor to his now well appreciated organizational skills. He was appointed the first chair of the newly formed graduate admissions committee, an enthusiastic recruiter of talented students to the programs and the principal investigator on one of the first NIH training grants under this new umbrella. Roy Vagelos commented, “Bradshaw was an enthusiastic and very effective recruiter of graduate students to Washington University. When the DBBS faculty decided that the University should have greater diversity among its graduate students, Ralph led a small group of faculty who visited historically black colleges to recruit top students to join the graduate programs of Washington University.”

Ralph was and is an inveterate keeper of data and how it is organized, a master of the Excel sheet. Shortly after returning from a sabbatical in Australia, Ralph assembled a collage in his home study, where we hosted aspiring applicants to the program. The collage included dozens of memorabilia from his travels neatly arranged in order. I had just returned from a trip to the United Kingdom, and I had a train ticket stub in my pocket, which, as a joke, I slipped behind one of the items on Ralph's collage. Wouldn't you know it, but first thing the next morning, I got a telephone call: “Stahl, why are you messing with my (bleep) board?” Obviously, this man has an eye for order that carries throughout his life, his various collections of books and memorabilia, on through to his research and his editorial work.

Ralph has been an “includer,” striving to enhance representation of historically excluded groups, bringing scientists together to enhance collegiality, serving as an officer of multiple scientific societies and organizations. Here is a quote from Amy Bradshaw (Professor of Cell Biology at the Medical University of South Carolina): “Growing up, our house had many visitors from all over the world, which allowed me to see and appreciate the wonder of different perspectives—an ideal reinforced and practiced by both my parents. One fond childhood memory I have is our annual Thanksgiving feast. At Ralph and Penny's, we often had a large gathering. As many people in the department were international, there were frequently guests who had never heard of Thanksgiving or had only a vague notion of this American tradition. My mom would make an enormous turkey, and everyone would bring dishes from all over the world to complete the meal. I would be in charge of making place cards so that my father could arrange the seating around the “table,” which usually consisted of many tables pushed together. At an early age, I saw the diversity of people that science brings together. That science (and a love for good food) provides a common thread that most often comes with respect and acceptance for our differences, was a gem of a life lesson that I learned around that big Thanksgiving Day table.”

In 1982, Ralph moved to take up the Chair of Biological Chemistry at the University of California, Irvine, where he built a strong department recruiting new faculty to the program. During this time, Ralph made seminal discoveries through the sequencing, characterization, and functional elucidation of many essential proteins involved in cell signaling. His work on epidermal growth factor (EGF) and its prototypical signaling receptor, is exemplar. At that time, polypeptide growth factor camps were still divided between mitogenic versus neurotrophic growth factors, and Ralph's work broke these artificial walls by showing that mitogenic growth factors could in fact act as neurotrophic factors (and vice‐versa) depending on the cellular context and receptor involved. The signaling of neurotrophic factors in neuronal cells was also extensively deciphered by a combination of complementary methodological approaches. The Bradshaw lab became an internationally respected and inspiring reference in the field of cell signaling.

Hubert Hondermarck (Professor of Biochemistry, University of Newcastle, Callaghan, NSW, Australia) remarked, “Ralph Bradshaw was also among the few pioneers of the field of proteomics. Not only did he publish germane papers in the field, particularly in relation to cell signaling, and organize many international meetings, but his role was particularly determinant through the launch of the reference journal in the field: *Molecular and Cellular Proteomics* (*MCP*). The then emerging field of proteomics needed a flagship journal with high standards for publication of high‐quality proteomic data, and with his natural scientific rigor and energy, Ralph (together with Al Burlingame from the University of California, San Francisco) provided exactly that. It should be noticed that his guidelines for proteomic data publication are still the reference today and they have been implemented by most leading journals in life science.”

Ralph's interest in the general welfare of biomedical research led him to hold membership on no fewer than 22 editorial boards, including Editor in Chief of *Trends in Biochemical Sciences*, *Molecular Cell Biology Research Communications*, and *FASEB BioAdvances*. As mentioned above, Ralph served as founding editor of *MCP* and was an early supporter of the Keystone Symposia, as well as a founder and former president of The Protein Society. Among all of these various and overlapping activities, Ralph played a key role in the evolution and success of the Federation of American Societies for Experimental Biology (FASEB). Judy Bond (Professor of Biochemistry Emerita, Pennsylvania State University) writes, “Ralph has been a leader, a consensus builder, and extraordinary contributor to FASEB for over three decades. In the 1990s, he led Federal Funding Conferences that were influential in setting annual increases to biomedical agencies (e.g., NIH, NSF), and participated in these conferences for many years. He was part of the leadership of FASEB that organized a meeting of academic and industry leaders with members of the US House of Representatives that led to the doubling of the NIH budget over a 5‐year period, a substantial achievement for biomedical research! During his presidency of FASEB (1995) and beyond, he continued to enhance relations with members of Congress and was a fierce advocate of bipartisan support for biomedical research. He organized a consensus conference in 1996 that brought together a coalition of 40 biomedical organizations to advocate for the reasonable and responsible regulation of scientific misconduct, and agree on the identification of fabrication, falsification, and plagiarism as misconduct activities. He played a continuous influential role in the extraordinary growth of the Federation in the 21st century, and he became engaged in delving into the history of the organization. Ralph was the inspiration, creator, and author of the book documenting the history of the Federation, *The Federation of American Societies for Experimental Biology: A Century of Service and Advocacy* (Bethesda, MD: FASEB, 2019). The book documents in detail the growth, growing pains, accomplishments, and legacy of the Federation from its founding in 1912 to 2017. His tireless efforts on behalf of FASEB continued in the last few years as he served on the Board of Directors representing the US Human Proteome Organization, chaired a Task Force on Governance for the Future of FASEB, and served as Editor‐in‐Chief of *FASEB BioAdvances*.”

Ken Thomas (Cofounder and Advisor at Trefoil Therapeutics, San Diego, CA, USA) writes, “In addition to Ralph's numerous academic achievements and contributions, he has been actively involved in applying his extensive scientific expertise and perspective in protein biochemistry as an industrial consultant and member of corporate Scientific Advisory Boards, many of which he has chaired. Notably, he also has cofounded several companies, recently including, along with three of his former students, Trefoil Therapeutics, a clinical stage biotechnology company arising from early work in his lab on the discovery of the protein growth factor FGF‐1. Ralph has remained intimately involved in the development of Trefoil, serving as Chief Scientific Officer and currently as Scientific Advisory Board Chair. He has approached these entrepreneurial activities with integrity, knowledgeable insights, and enthusiasm, making working with him a consistently rewarding and enjoyable experience, not only as a cofounder but also an independent scientific collaborator and originally a postdoctoral fellow.”

Ralph Bradshaw, scientist, teacher, editor, and entrepreneur, is one of a group of individuals, scientists, and physician‐scientists, educated in the 1960s before entering the academy in the 1970s, who have helped transform and advance American life sciences to levels unanticipated even by the most sanguine observer. This group of individuals has contributed to and supported graduate and medical education; they have been key drivers of the expansion of our basic understanding of molecular processes in cells and organisms; they have widely contributed to the quality and mode of communication that links scientists and their communities with the public; and they have catalyzed the transfer of basic science to the applied arena, from bench to bedside. These attributions are reflected in the careers of this group of senior scientists. Now aging, some of this cohort have excelled in one or more of these areas of excellence. A smaller number have made their mark in all of the above. This is the category that represents the contributions by Ralph Bradshaw, for which we owe a significant debt of gratitude and admiration.

